# Acute Traumatic Pain in the Emergency Department

**DOI:** 10.3390/diseases11010045

**Published:** 2023-03-03

**Authors:** Christian Zanza, Tatsiana Romenskaya, Marta Zuliani, Fabio Piccolella, Maria Bottinelli, Giorgia Caputo, Eduardo Rocca, Antonio Maconi, Gabriele Savioli, Yaroslava Longhitano

**Affiliations:** 1 Department of Integrated Research and Innovation Activities, Unit of Translational Medicine AON SS, Antonio e Biagio e Cesare Arrigo H, 15121 Alessandria, Italy; 2 Department of Physiology and Pharmacology, Sapienza University of Rome, 00185 Rome, Italy; 3 Emergency Medicine and Surgery, IRCCS Fondazione Policlinico San Matteo, 27100 Pavia, Italy; 4 Department of Anesthesiology and Perioperative Medicine, University of Pittsburgh, Pittsburgh, PA 15260, USA

**Keywords:** emergency, traumatic pain, pain management, trauma management, acute pain

## Abstract

Trauma is a major cause of mortality throughout the world. Traumatic pain—acute, sudden, or chronic—is defined as “an unpleasant sensory and emotional experience associated with actual or potential tissue damage”. Patients’ perceptions of pain assessment and management have become an important criterion and relevant outcome measure for healthcare institutions. Several studies show that 60–70% of ER patients experience pain, and more than half of them express a feeling of sorrow, which can be moderate or severe, at triage. The few studies that have analyzed how pain is assessed and managed in these departments agree that approximately 70% of patients receive no analgesia or receive it with remarkable delay. Specifically, less than half of the patients receive treatment for pain during admission and 60% of discharged patients have higher intensity pain than at admission. Trauma patients are also the ones who most commonly report low satisfaction with pain management. Associated with this lack of satisfaction, we can describe the poor use of tools for measuring and recording pain, poor communication among caregivers, inadequate training in pain assessment and management, and widespread misconceptions among nurses about the reliability of patients’ estimation of pain. The aim of this article is to review the scientific literature to explore the methodologies of pain management in trauma patients attending the emergency room and analyzing their weaknesses as a starting point to improve the approach to this, unfortunately too often, underestimated issue. A literature search was performed using the major databases to identify relevant studies in indexed scientific journals. The literature showed that the multimodal approach in trauma patients is the best approach to pain management. It is becoming increasingly crucial to manage the patient on multiple fronts. Drugs acting on different pathways can be administered together at lower doses, minimizing risks. Every emergency department must have staff trained in the assessment and immediate management of pain symptoms as this allows the reduction of mortality and morbidity and shortens hospital stays, contributing to early mobilization, reduced hospital costs, and enhanced patient satisfaction and quality of life.

## 1. Background

Trauma is a major cause of mortality throughout the world. In recent years, major advances have been made in the [1] management of trauma. The result of these has been a reduction in mortality and an improvement in patients’ distress experience. Clinicians and researchers have a particular interest in pain control [2]. The improvement of pain management not only results in greater comfort for trauma patients, but also reduced the stress response, decreased systemic opioid side effects, and reduced chronic pain. Pain relief is one of the most prevalent requests among trauma patients in emergency rooms [3]. These types of patients also frequently complain of not being cared for enough in terms of pain control [4]. Pain control leads to the decrease of stress response effects, such as tachycardia with subsequent myocardial ischemia or fatal congestive heart failure. As an example of this, we cite the Matot et al. study that compared the effect of epidural analgesia versus standard analgesia (intramuscular opioids) in the initial preoperative period in high-risk cardiac patients with hip fractures. Preoperative adverse cardiac events were significantly more prevalent in the control group compared with the epidural group (7 of 34 vs. 0 of 34; *p* = 0.01) [5].

In recent years, the management of trauma patients has been one of the most resource-intensive aspects of medical care performed in emergency department settings with limited resources [6,7]. Trauma patients include a wide spectrum of physiologically varied patient populations, including healthy youngsters, children, and the elderly.

The characteristics of patients must be considered to provide an accurate diagnosis, including the main factors influencing injury risk, such as age and gender [8,9,10]. It has been reported, for example, that ankle distortions and knee injuries are more frequently seen in women, probably due to higher estrogen levels, greater fat mass, lower muscle mass, higher flexibility, and wider pelvis [11]. At the same time, chronic and overuse injuries are more common in males due to the loss of elasticity and less effective repair mechanisms of tendons and muscles [12,13].

In ER situations, acute traumatic injuries are mostly characterized by sudden trauma to the tissue, commonly related to a specific identifiable event. Acute-related injuries can be classified according to the tissues involved in the injury (e.g., bone, ligament, muscle, tendon, joint) and the type of injury (e.g., fracture, dislocation, sprain, or strain) [14].

Several studies in the literature show that the inappropriate treatment of pain is still a common phenomenon in the management of the early stages of trauma, both in out-of-hospital and in-hospital environments. Unfortunately, in the case of inadequate pain control, patients, especially those with a traumatic incident, can require invasive anesthesiologic procedures, such as general anesthesia with intubation. Another most feared consequence may be post-traumatic stress disorder (PTSD) [15]. An extremely well-represented study was carried out in Canada on a population of pediatric patients. In this case, the prevalence of PTSD in patients with trauma ranged from 7 to 40%. About 50% of patients with PTSD after an automobile crash required treatment for chronic pain. Moreover, this critical post-traumatic complication can persist for years and could contribute to morbidity or lead to a depressive syndrome.

The culpability for inadequate pain control can be ascribed to cultural, professional and organizational failures. Specifically, these include a lack of concern for the patient’s distress and inappropriate drug use (insufficient knowledge of indications, contraindications, side effects, complications, synergies). Furthermore, there is insufficient interdisciplinary organization and collaboration, and there is no systematic application of a pain score (for example, Visual Analogue Scale (VAS) [15].

The analgesic pharmacological approach historically represents the cornerstone of pain management for acute traumatic injuries [3,16]. The pharmacological approach is only one component of an integrated, multidisciplinary approach aimed at reducing physical impairment, optimizing functional recovery, and improving the patient experience [17,18]. The IOC consensus statement [19] recommends a medical prescription tailored to the patient. The goal is to minimize side effects and achieve the maximum pain relief outcome. The type of tissue involved, type of injury, and severity are critical in defining the most appropriate treatment approach [11,13,20,21,22].

The objective of our narrative review is to explore the literature and to report the key strategies for pain treatment in trauma patients approaching the Emergency room in a unique paper.

## 2. Material and Methods

A literature search was performed to identify relevant studies in indexed scientific journals using the following major databases: PubMed, MEDLINE, EMBASE, and the Cochrane Controlled Clinical trials register. The following search terms were used: trauma, pain, traumatic injuries, pain management, ER department, and drugs. The search was filtered for human studies, and the publication period was set from 2010 to 2022. The period was chosen to focus on recent publications given the topic breadth. We excluded editorials, commentaries, letters to the editor, opinion articles, meeting abstracts, and original articles lacking an abstract. Research was limited to clinical trials, meta-analyses, randomized controlled trials (RCT), reviews, and systematic reviews. Search criteria included the following: “pain in ER”, “traumatic pain”, “pharmacology pain management”, “Pain Management”, “Trauma management”, “ER pain relievers”, “trauma consequences”.

## 3. Discussion

### 3.1. Evaluation of Signs and Symptoms

The World Health Organization (WHO) pain scale was developed in 1986 as an abstract model to guide cancer pain intervention [23].

Pain produces a physiological stress response that includes increased heart and breathing rates to facilitate the increasing demands of oxygen and other nutrients to vital organs. Failure to relieve pain produces a prolonged stress state, which can result in harmful multisystem effects. Despite this, we focused on acute traumatic pain that can often be resolved with symptomatic and time-limited expedients. Ineffective pain management can lead to a marked decrease in desirable clinical and psychological outcomes and patients’ overall quality of life. Effective management of acute pain results in improved patient outcomes and increased patient satisfaction.

Pain is one of the most common reasons for patients to visit the emergency department, so we focused on the management of this period. Due to the extensive number of visits to the ED related to pain, emergency medicine physicians should be experts in providing safe, effective, and timely pain management.

Pain assessment of trauma patients tin the Emergency room may not be at all easy for various reasons, including the patient’s age, emotional condition (anxiety, psychomotor distress), facial trauma, and loss of consciousness. Thus, over the years, several standardized unidimensional Pain Scales have been studied for the assessment of acute pain. Table 1 shows the most used rating scales according to age.

Today there is worldwide agreement promoting the use of a pain scale for medical interventions for all pain associated with serious illness, including chronic trauma pain [8,10,24,25]. Trauma patients usually claim severe pain, scoring 7–10/10 on the VAS scale.

The pain can be classified as mild-to-moderate pain (VAS 1–3) that is responsive to Paracetamol and/or NSAIDs; moderate-to severe pain (VAS 4–6) responsive to soft opioids and/or NSAIDs and Paracetamol; and severe pain (VAS 7–10) requiring treatment with strong opioids and NSAIDs.

The scientific literature recommends administering fast-acting agents intravenously in small boluses at frequent intervals until a tolerable pain condition is maintained.

Starting from this, the physicians have a baseline to structure long-term analgesia. It is not possible to expect to achieve perfect analgesia. Co-administration of drugs, whether by the same or different ways, allows an excellent result with minimal risk.

Systemic routes are the most suitable options in the emergency phase of a trauma case. Intravenous titration of small boluses also allows adaptation to individual variations. Intramuscular or subcutaneous injection of opioids is generally not as effective and will have to be performed with extreme care in the presence of hypovolemia.

### 3.2. Pain Managment

Patients with acute trauma frequently require complex management with coexisting, often competing, priorities. For the sake of clarity, let us underline the definitions of the possible actions that can be used when talking about anesthesia. Local anesthesia: the anesthetic drug is injected into the skin in order to anesthetize a small area, usually superficial. Regional anesthesia: the two most common loco-regional anesthesia approaches are spinal and epidural, which are useful for anesthetizing large regions of the body, for example, from the waist down. In many cases, the patient remains awake and conscious with no pain, and there may be reduced or absent tenderness to the area to be operated on.

General anesthesia (also called “total” anesthesia): in this approach, the patient loses consciousness; both inhalant and intravenous drugs may be used. Recall, then, the possibility of using only superficial or deep sedation, which allows the treatment of anxiety and pain by inducing a light sleep-like state in the patient, but which can be easily interrupted by “waking” the subject if necessary. High-quality analgesia in the trauma population must also be addressed. In addition to improving patient comfort, peripheral nerve and neuraxial block as well as nonopioid agents significantly reduce the requirement for systemic opioid analgesia and the adverse effects associated with opioid use. Moreover, all these help to prevent the development of long-term sequelae. This is often critical in the multiply injured patient who suffers from neurologic, cardiovascular, and/or pulmonary impairment. In addition, the early use of regional anesthetic techniques in selected trauma patients appears to improve outcomes such as pulmonary morbidity, delirium, and mortality and facilitates reductions in the overall length of stay in both the emergency room and hospital.

Absolute contraindications must be considered for tailored analgesia, which include:

- Patient refusal.
- Infection at the injection site. The insertion of a needle through infected into healthy tissue may spread infection. In addition, local anesthetics do not work well in acidotic tissue.
- An allergy to local anesthetics.
- The inability to guarantee sterile equipment to perform the block. This is an absolute contraindication as this could result in the introduction of infectious agents into otherwise healthy tissue.
- Great risk of local anesthetic toxicity (i.e., one would not want to perform or repeat a bilateral axillary block).

#### 3.2.1. Administration Routes for Trauma Patients in the Emergency Department

Choosing the most effective route for administering analgesia is a crucial moment in pain management of trauma patients. Since these kinds of patients must remain with an empty stomach in the planning for possible surgery, the oral route is not advisable. Subcutaneous/intramuscular administration is also not recommended in this type of patient since the absorption of drugs depends on local vasculature, which is often poor.

The rectal route may be considered in patients who are unconscious, unable to swallow, or those who must remain fasting.

Some opioid drugs, such as fentanyl or buprenorphine, can be given by the transdermal route. Attention should be paid to the fact that by the time the analgesic patch is removed, a subcutaneous reservoir remains on the skin that will continue releasing the opioid into the circulation; although in minor concentrations, this can lead to respiratory depression.

The terminus of intraspinal analgesia as a route of analgesia administration includes either epidural or subarachnoid injection. Drugs are deposited in the corresponding spaces, epidural space, and subarachnoid space. Despite the instant benefit, these routes of drug administration are not widely used in acute pain management in the ED.

Based on the above, it can be deduced that the most widely used route in acute pain control in trauma patients is the intravenous route. This approach allows different drugs to be administered, such as non-opioid agents, steroids, nonsteroidal anti-inflammatory drugs or opioids. Opioids can be administered via PCA (patient-controlled analgesia). This is one method for managing moderate to severe postoperative pain, especially among patients who cannot take oral medications [26].

#### 3.2.2. Pharmacological Approach

The *pharmacological approach* is one of the most used in emergency rooms for pain management in trauma patients. The ideal drug would be rapid-acting drugs with minimal side effects. We have listed below the most recommended drugs in pain management.

*Paracetamol*, or acetaminophen, is the most used medication and is usually used for mild/moderate pain [9,11,20]. The molecule can be used alone or combined with different active excipients. The main recommendations cite paracetamol as the first line for the treatment of moderate pain [16,26].

The constant increase in chronic conditions in hospital patient populations makes it necessary to focus on pain management strategies that are as safe as possible. Ideally, analgesic drugs should not interfere with or exacerbate chronic syndromes such as renal failure, coronary artery disease, and heart failure, and should not interfere with concomitant treatment, including with antiplatelet drugs. In this regard, recent research has clearly shown that there is a strong association between NSAID use and increased mortality, especially in patients with severe trauma [10,19]. The combination of paracetamol with codeine is equivalent to ketorolac in terms of pain control efficacy, mostly in the case of patients with post-traumatic pain. This association is also superior in acute pain and in patients with fractures and muscle pain.

The use of *NSAIDs* for pain management is highly developed and supported by a large amount of literature [10,27]. The reasons are to be found in the easy means of administration and easy association. NSAIDs exist in several forms, as topical gels, foams, and dermatological creams, oral tablets and capsules, in transdermal absorption systems (commonly referred to as medicated patches), or in suppositories. The use of diclofenac and ketoprofen is typical, and when applied topically on acute sprains and strains, they relieve 50% of pain after one week of use, according to a recent Cochrane review [28]. To date, ketorolac is the most widely supported NSAID in the management of acute pain, as confirmed by a recent meta-analysis that highlights the role of ketorolac in the relief of severe pain, and it has an adequate safety profile when discharged in 5 days [29]. They are prescribed as general anti-inflammatory pain relievers to decrease inflammation in musculoskeletal, rheumatologic, joint, and similar conditions, as well as post-surgery. Differences in NSAID anti-inflammatory activities are modest but can result in considerable variability in individual patient responses.

Italy is among the top countries in the world for the inappropriate and too-frequent use of nonsteroidal anti-inflammatory drugs, according to the OsMed Report 2020 [30].

NSAIDs are the most prescribed analgesic drugs in the world, and their efficacy in the treatment of acute pain has been repeatedly demonstrated [4,29,31,32].

Their action is based on inhibition of the reversible cyclooxygenase enzyme that mediates the production of prostaglandins and thromboxane A2 [4]. Prostaglandins are involved in various physiological functions, such as the maintenance of gastric mucosal integrity, regulation of kidney blood flow and regulation of endothelial tone; they also play a key role in inflammatory and nociceptive processes. Indicated in mild and moderate pain, they are limited by the “ceiling effect”; maximum efficacy cannot be further improved by increasing doses [33,34,35,36].

They are burdened with major side effects: gastrointestinal bleeding, hepatic and renal toxicity, asthma, and inhibition of platelet aggregation [13]. There are specific health issues that should be considered as contraindications, such as the presence of a hemorrhagic focus (severe fractures) and/or the indication and possibility of an early surgical intervention. Safe NSAID use requires an assessment of the hemorrhagic and CV risk to the patient, not always an easy task, and a strict evaluation of the relationship between adverse events and expected benefits [19]. For instance, drugs with greater selectivity for COX2 provide greater gastric tolerance and do not interfere with the antiplatelet function of ASA but may significantly increase CV risk [5].

The administration of NSAIDs in patients with nephropathy treated with ACE-inhibitors, diuretics or anti-coagulant therapy should be avoided and requires close monitoring of renal function and the hemocoagulative status.

NSAIDs should be avoided in the first 72 h after concussion due to increased risk of hemorrhage [13,16,19,23,34,35]. Furthermore, inflammation plays a crucial role in the early processes underlying tissue healing in the musculoskeletal system [36] so the anti-inflammatory pharmacological approach has recently been questioned [30].

*Opioids* are indicated in the presence or prediction of severe pain (VAS 7–10). Morphine is the parenteral drug. Its characteristics make it safe and effective. [30]. Furthermore, in the case of overdose, a specific antidote is available. Clinical effects last about six hours; it is advisable to continue thereafter with adequate continuous infusion.

The theoretical total/day dosage is not related to body weight, but to age: mg = 100 − age in years (excluding extreme ages); however, individual sensitivity can be different and is genetically determined. These drugs should be prescribed for major traumas that could be related to a high risk of bleeding and severe pain. Indeed, in these cases, NSAIDs do not represent a suitable option, according to the WHO pain ladder recommendations [37,38]. In this context, the NICE guideline for major trauma in adults [38] supported the use of intravenous morphine as the first-line analgesic in hospital or pre-hospital settings to achieve adequate pain relief without varying blood coagulation parameters. It has been recommended that opioids should be considered for a period not exceeding 5 days [39]. Longer prescriptions have been reported despite the critical issue of opioid dependence or addiction.

*Ketamine* is a non-opioid analgesic that has been administered in the clinic for decades. This drug is a strong analgesic and affects the sympathetic nervous system, which can benefit trauma patients [40]. For instance, ketamine increases the average heart rate and blood pressure, which are both favorable for stabilizing the trauma patient’s condition [41,42]. Furthermore, ketamine does not have the opioid’s dangerous side effects and does not affect the patient’s respiration in any way. Thus, ketamine could be considered as a proper substitute for opioid analgesics. In recent years, many clinical trials have been conducted indicating that ketamine can be beneficial in the pain management of trauma patients in prehospital settings. As an example, in 2014, Tran et al. demonstrated that ketamine has analgesic effects equivalent to morphine, while the risk of its respiratory side effects is lower than morphine. Nonetheless, delusion and agitation are other side effects of ketamine [42].

#### 3.2.3. Invasive Pain Management in the Emergency Room

Regional anesthesia (RA) has all the characteristics of being a first-order analgesic technique [4,6]. Moreover, new innovative techniques, such as ultrasound [43,44], permit its use more safely and limit the risks of blinding procedures [6]. Mini-invasive techniques are commonly used in clinical practice for chronic pain [35,36], but they are still not adequately studied and performed for acute traumatic injuries [37,38,39].

The regional analgesia provided through an epidural or brachial catheter should be considered for all trauma patients; this approach can, potentially, spare the use of systemic opioids and facilitate early mobilization. Epidural analgesia has been shown to produce high levels of patient satisfaction and improve lung function after thoracoabdominal and major orthopedic surgery in elective populations [40]. We retrieve similar benefits in trauma patients. However, regional techniques are less practical when the patient has multiple injury sites, fractures, or open wounds. Epidural placement in anesthetized patients has a relative contraindication due to potential occult spinal cord injury (SCI). The risk–benefit ratio supports positioning during surgery when the general anesthesia helps patient positioning and cooperation [41].

The treatment must be tailored to the injury site. In this regard, upper forearm nerve blocks or axillary blocks, in particular, may be effective in the pain management of hand and forearm fractures. Furthermore, management of distal radius fractures during reduction could involve blocking the hematoma (getting good results in pain reduction). Likewise, blocking the sciatic nerve, adductor canal, iliac fascia, and femur have been shown to be effective in specific pathologies to achieve adequate analgesia and reduce opioid medications after acute traumatic injuries requiring surgery [28,42,43,44]. Despite the promising effects of nerve block injections, some authors have reported that regional anesthesia could mask the onset of compartment syndrome, so these procedures should be performed with due care [29].

However, it should be noted that although nerve block may improve pain management and reduce oral drug administration, multimodal analgesia has been strongly recommended. Thus, minimally invasive procedures should be considered in a combination of pharmacological strategies to potentially reduce the side effects of monotherapy and improve pain management in acute injuries [36].

Regional anesthesia has an important role in planning a multimodal strategy to minimize nociceptive responses and sequelae [42]. Future research on regional anesthesia in trauma patients in the emergency room should not only be limited to the isolated efficacy of a particular technique or its impact on pain control but should also include a consideration of its contribution in the context of overall analgesic management.

Conscious sedation of patients who receive regional anesthesia facilitates the procedure by reducing anxiety, pain and noxious stimuli while allowing maintenance of independent airway control. In addition, this method keeps the protective reflex, stabilizes vital signs and better promotes recovery. Conscious sedation is much more safe than deep sedation because the complications around the operation are rare compared to deep sedation, which has incidence rates ranging from 25–75% [45,46]. Therefore, conscious sedation is appropriate for patients during regional anesthesia. Mingus et al. [45] reported that while remifentanil used as a TCI method had better analgesic effects without excessive sedation than propofol, it had more frequent side effects, including respiratory depression, nausea and vomiting, and they recommended dose reduction for elimination of these side effects.

We submit a series of nerve blocks that can be widely used by all emergency specialists. The guidelines suggest contacting the orthopedic department as soon as possible for any patients who need to be admitted or who will require consultation in the ED, but do not delay standard analgesia or regional analgesia for:

- Dislocation of the shoulder.
- Fracture of the clavicle.
- Proximal humerus fracture.
- Low-energy distal radius fracture.
- Hand and finger injuries.
- Fracture and dislocation of the hip.
- Low-energy fractures of the foot and ankle.

Obviously, in doubtful situations, it is essential to wait for the anesthesiologist specialist or a physician with more experience in practice.

Here again, we emphasize how collaboration between specialists allows a higher standard of care and, overall, limits overcrowding [47,48].

## 4. Conclusions

In this review, an attempt has been made to summarize the most important information about pain management in the trauma patient (Table 2). We focused on methods that should be as fast, safe, and effective as possible so that they can be easily used in the ER. In addition to the drug or technique, we would like to underline how the literature is shifting its focus to the multimodal approach. It is becoming crucial to manage patients’ issues on multiple fronts. Drugs, when administered together, can be used at lower doses, acting on different pathways and minimizing risks. Further research is needed to make clear the impact of peripheral nerve blocks and neuraxial analgesia on outcomes such as the development of delirium, mobility, chronic post-traumatic pain, and post-traumatic stress disorder. While peripheral nerve blocks may not delay the diagnosis of acute compartment syndrome, prudent use of regional techniques in trauma patients should be combined with a multidisciplinary approach, astute clinical judgment, and vigilance.

Every emergency department must have trained staff for an effective and immediate management of pain symptoms. The refinement of this multimodal approach will allow the reduction of mortality and morbidity and shorten hospital stays, contributing to early mobilization, reduced hospital costs, and enhanced patient satisfaction and quality of life.

## Figures and Tables

**Table 1:** The main pain rating scales in the different populations [2].

Age	Scale	Rating	Interpretation
***age 0–4 years old***	***FLACC*** Faces, Arms, Legs, Cry, Consolability	0–2 points for each domain. Maximum score will be between 0–10.	0 relaxed and comfortable 1–3 Mild discomfort 4–6 Moderate pain 7–10 Severe discomfort/pain
	***CHEOPS*** *Children’s Hospital of Eastern Ontario Pain Scale* (cry, facial, verbal, torso, touch, legs)	0–3 points for each domain.	>8 points moderate pain, which requires treatment
***age 4–10 years old***	***FPS*** *Faces Pain Scale*	The scale uses the association of stylized facial expressions.	scores from 5 to 1 on a decreasing pain scale
	***Faces Pain Scale—Revised*** the age range 4–16 years	Score the chosen face 0, 2, 4, 6, 8, or 10, counting left to right.	“0” equals “No pain” and “10” equals “Very much pain”
	***Wong–Baker FACES^®®^*** Six faces, corresponding to six different degrees of pain	Each face also has a number from 0 to 10, which coincides with the intensity of pain.	“0” equals “No pain” and “10” equals “Very much pain”
***age >10 years old/adult***	***VRS*** *Verbal Rating Scale*	The patient verbally rates the pain.	0–4: none, mild, moderate, severe, intolerable
	***VAS*** *Visual Analogue Scale*	A 10 cm long straight line whose extremes represent the conditions of no pain and maximum possible pain.	
	***NRS*** *Numerical Rating Scale*	Scale of pain intensity from 0 (no pain) to 10 (the most terrible pain imaginable).	

**Table 2:** Summary of the most important articles in the narrative review.

Title	Period	Population	Conclusion
**Cordell, WH et al.** *The high prevalence of pain in emergency medical care. The American Journal of Emergency Medicine* [1].	2022	ED patients (aching, burning, and discomfort).	Pain was a chief complaint for 52.2% of the visits.
**Todd, KH et al.** *A Review of Current and Emerging Approaches to Pain Management in the Emergency Department* [2].	Systematic review—2000–2018		A new generation of emergency physicians is seeking additional training in pain medicine to obtain a better management of pain in the emergency department.
**Court-Brown, CM et al.** *The epidemiology of acute sports-related fractures in adults* [3].	Retrospective analysis—2000	Database containing all in-patient and out-patient fractures in a defined patient population in 2000.	Sporting activities are the third most common cause of fractures and traumatic pain.
**Raffa, RB et al.** *Pharmacology of oral combination analgesics: Rational therapy for pain* [13].			Many combination analgesics are available and are commonly prescribed for pain. The goal is to facilitate patient compliance, simplify prescribing and improve efficacy without increasing adverse effects.
**Xia, AD et al.** *Evaluation of pain relief treatment and timelines in emergency care in six European countries and Australia* [18].	Observational, retrospective chart review—2013–2017	Treatment outcomes in medical emergency situations in Sweden and Australia.	While effective pain management is an important part of emergency care, oligoanalgesia is frequently reported and can have a substantial impact on patients’ physical and emotional wellbeing.
**Lyrtzis, C et al.** *Efficacy of paracetamol versus diclofenac for Grade II ankle sprains* [21].	Controlled randomized study—2011	Ninety patients, 18 to 60 years old, with Grade II acute ankle traumatic sprains.	Diclofenac and paracetamol had the same effect on pain reduction.
**Craig, M et al.** *Randomized comparison of intravenous paracetamol and intravenous morphine for acute traumatic limb pain in the emergency department* [10].	Randomized, double-blind pilot study—2012	Patients between 16 and 65 years old with isolated limb trauma and in moderate to severe pain.	Intravenous paracetamol appears to provide a level of analgesia comparable to intravenous morphine.
**Abdollahpour, A et al.** *A review on the recent application of ketamine in management of anesthesia, pain, and health care* [32].	Systematic review—2020		Ketamine is a drug of choice for the cases where opioid tolerance, inflammatory pain, neuropathic pain component, and depression or a combination of these factors are problematic.
**Diwan, S et al.** *A retrospective study comparing analgesic efficacy of ultrasound-guided serratus anterior plane block versus intravenous fentanyl infusion in patients with multiple rib fractures* [39].	Retrospective cohort study—2021	72 patients in ED for multiple rib fractures.	US-guided SAPB is an opioid-sparing, effective interfacial plane block which is safe and should be considered early in all patients.
**Gadsden, J et al.** *Regional anesthesia for the trauma patient: improving patient outcomes* [40].	Systematic review—2015		RA in the ED is a valuable, opioid-sparing tool in multimodal pain control with a positive impact on patient LOS and some traumatic injury outcomes.

## Data Availability

Not applicable.
